# Robust Hand Gesture Recognition Using a Deformable Dual-Stream Fusion Network Based on CNN-TCN for FMCW Radar

**DOI:** 10.3390/s23208570

**Published:** 2023-10-19

**Authors:** Meiyi Zhu, Chaoyi Zhang, Jianquan Wang, Lei Sun, Meixia Fu

**Affiliations:** 1 School of Automation and Electrical Engineering, University of Science and Technology Beijing, Beijing 100083, China; m202120747@xs.ustb.edu.cn (M.Z.); wangjianquan@ustb.edu.cn (J.W.); sun_lei@ustb.edu.cn (L.S.); mxfu1205@ustb.edu.cn (M.F.); 2 Key Laboratory of Knowledge Automation for Industrial Processes of Ministry of Education, University of Science and Technology Beijing, Beijing 100083, China

**Keywords:** millimeter-wave sensors, frequency-modulated continuous-wave radar, deep learning, gesture recognition

## Abstract

Hand Gesture Recognition (HGR) using Frequency Modulated Continuous Wave (FMCW) radars is difficult because of the inherent variability and ambiguity caused by individual habits and environmental differences. This paper proposes a deformable dual-stream fusion network based on CNN-TCN (DDF-CT) to solve this problem. First, we extract range, Doppler, and angle information from radar signals with the Fast Fourier Transform to produce range-time (RT) and range-angle (RA) maps. Then, we reduce the noise of the feature map. Subsequently, the RAM sequence (RAMS) is generated by temporally organizing the RAMs, which captures a target’s range and velocity characteristics at each time point while preserving the temporal feature information. To improve the accuracy and consistency of gesture recognition, DDF-CT incorporates deformable convolution and inter-frame attention mechanisms, which enhance the extraction of spatial features and the learning of temporal relationships. The experimental results show that our method achieves an accuracy of 98.61%, and even when tested in a novel environment, it still achieves an accuracy of 97.22%. Due to its robust performance, our method is significantly superior to other existing HGR approaches.

## 1. Introduction

In recent years, computer technology has penetrated all aspects of our daily life, and human–computer interaction has become ubiquitous. With the development of wireless sensor technology, people are beginning to pursue more natural and convenient approaches to human–computer interaction, such as gesture recognition. As an emerging human–computer interaction technology, gesture recognition can be widely used in the Internet of Things (IoT) [1], smart home systems [2], robot control [3], and VR [4] games. Most proven techniques for gesture recognition are based on vision [5,6] and wearable sensors [7,8]. Gesture recognition techniques based on wearable sensors collect motion information by analyzing the signals from devices such as accelerometers and gyroscopes. Nevertheless, these techniques require users to wear additional sensors on their hands, which is an uncomfortable experience. Gesture recognition techniques based on computer vision generally obtain the RGB or depth image of the gesture through the camera, preprocesses the image, and then extracts the image features for classification and recognition using deep learning and other methods. However, these techniques have some deficiencies. Video images may contain a substantial amount of personal data, posing a potential risk of information leakage, and the recognition is susceptible to environmental conditions such as lighting changes and obstructions.

Gesture recognition techniques based on millimeter-wave frequency-modulated continuous wave (FMCW) radars effectively overcome the limitations of wearable devices and computer vision-based methods [9,10,11]. Notably, the utilization of higher radio frequencies facilitates the design of more compact sensors, making it feasible to incorporate them into smaller devices. Additionally, the 77 GHz FMCW radar, with its impressive bandwidth of up to 4 GHz and a shorter wavelength, markedly enhances the resolution in both range and angle measurements. Moreover, an added advantage of the millimeter-wave radar is its ability to safeguard user privacy and is not affected by changes in environmental light.

In summary, gesture recognition techniques based on 77 GHz FMCW millimeter-wave radars can protect user privacy and are not easily affected by environmental conditions, and they are characterized by a simple structure and low cost. This is a research topic worthy of attention. For instance, Google [12] used a 60 GHZ Soli radar to capture gesture data and proposed an end-to-end training combination based on deep convolutional and recurrent neural networks, which achieved a recognition rate of 87%. In RadarNet [13], a large-scale dataset consisting of 558,000 gesture samples and 3,920,000 negative samples was created to train the model and improve the algorithm’s robustness.

However, the existing gesture recognition methods have three limitations.

(1) Low robustness and environmental sensitivity. Traditional methods usually collect data in interference-free environments and do not adequately consider the impact of environmental interference and the ambiguity and variability of different users’ gesture expressions to be recognized. In addition, radars are sensitive to variations in body position and distance, which may also negatively affect the recognition performance.

(2) Inadequate utilization of data streams. Most existing studies focus primarily on investigating time–frequency maps (TFM) and spectrum map sequences (SMS) features separately. Such practice ignores the synergistic benefits between TFM and SMS, hindering a comprehensive understanding of gesture recognition.

(3) GPU underutilization. Recurrent neural networks (RNNs) are often used in the previous studies. However, the parallel computing power of the graphics processing unit (GPU) cannot be fully utilized to improve the computational efficiency.

To address the aforementioned limitations, we have prioritized the use of deep learning networks. Due to its successful application in computer vision [14], deep learning has been used as a classifier in millimeter-wave radar-based gesture recognition systems.

Initially, many researchers extracted the micro-Doppler features of gestures and used machine learning algorithms such as hidden Markov models (HMMs) and k-nearest neighbors (KNN) classifiers for gesture recognition and classification. Malysa G. et al. [15] measured the micro-Doppler features of six gestures using a 77 GHz radar, constructed images with spatially varying energy distributions of micro-Doppler velocities over time, and classified the gestures using an implicit Markov model. G. Li et al. [16] obtained the sparse radar echoes of dynamic gestures using a Gaussian window Fourier function and extracted micro-Doppler features using an orthogonal matching tracking (OMP) algorithm. Then, they combined the KNN classifier with the modified Hausdorff distance to identify these sparse micro-Doppler features. Ryu et al. [17] first generated RDMs from the FMCW radar’s raw signals and then extracted various features from these maps. They combined a feature selection algorithm and a quantum-inspired evolutionary algorithm (QEA) to identify the most relevant features for gesture recognition. Finally, they classified the gestures based on the selected feature subset. The aforementioned studies show that, although traditional machine learning methods are effective, they require manual feature selection and are labor-intensive, subjective, and highly dependent on a priori knowledge. In contrast, deep learning (DL) approaches allow neural networks to learn and extract features independently, making it easy to build end-to-end learning frameworks. Such approaches improve the accuracy of gesture recognition and enable real-time gesture recognition. Zhu et al. [18] considered radar spectrograms as a multi-channel time series and proposed a DL model consisting of one-dimensional convolutional neural networks (1D-CNNs) and long short-term memory (LSTM). Chen and Ye [19] proposed an end-to-end 1D-CNN with inception dense blocks, which uses the original radar echo sequence as an input to improve the speed of forward propagation. The temporal features of adjacent frames are extracted through 1D convolutions, and the global temporal information is processed by LSTM. One of the significant advantages of directly feeding raw radar signals into 1D-CNNs is the small number of parameters required. However, the inability to use radar signal processing algorithms to eliminate interferences limits the applicability of this method. Choi J. W. et al. [20] used Google’s Soli radar to capture gesture data and generate RDMs without clutter. The machine learning component included an LSTM encoder for learning the temporal properties of RDM sequences. Wang L. et al. [21] used a 340 GHz terahertz radar for gesture recognition, which can leverage the high precision provided by the terahertz frequency band to obtain accurate range-time maps (RTMs). These RTMs were then used in an intent model to interpret the intentions behind the gestures. Wang Y. [22] measured the range, Doppler, and angle information of gestures using fast Fourier transform (FFT) and MUSIC algorithms and obtained RTMs, DTMs, and angle-time maps (ATMs). Then, they used an algorithm combining residual learning with skip connections to extract detailed features from three-dimensional gesture maps. S. Hazra and A. Santra [23] constructed the time series of multi-feature spectrograms from RTMs and range-angle maps (RAMs) using FFT. The features were learned through a 2D-CNN, and an attention mechanism was employed to suppress distractions and extract useful gesture information. Finally, the gesture information was passed to LSTM layers for time modeling and classification. Yan B. et al. [24] conducted many experiments to compare the levels of effectiveness of RDMs, RAMs, Doppler angle maps (DAMs), and Micro-Doppler spectrograms in gesture recognition. Their conclusion is that RAMs have a significant advantage over other heat maps in gesture recognition under cross-user conditions. Wang Y. et al. [25] employed range-Doppler maps (RDMs) and range-angle maps (RAMs) as feature maps, which were fed into a dual 3D convolutional neural network for feature extraction and classification. Gan et al. [26] gathered echo data using a 24 GHz radar, extracted the range and Doppler information of gestures, and input the data into a 3D CNN-LSTM for gesture classification. These studies provide valuable insights by focusing on individual TFM or SMS features, but the inherent interconnections within these features are only partially captured due to the separate treatment of each features, which limits the scope for a more holistic and deeper understanding of gesture recognition. Additionally, some studies attempt to integrate TFM and SMS features. Wang Y. et al. [27] mapped a gesture action into 32 frames of RDMs and ATMs and then used LSTM to fuse the features. Yang Z. et al. [28] used the Discrete Fourier Transform (DFT), Multiple Signal Classification (MUSIC), and Kalman filter to extract the range-Doppler-angle trajectory of gestures appearing on the hypothetical gesture desktop. Meanwhile, they designed an LSTM network that utilizes a repetitive forward propagation method to incorporate spatial, temporal, and Doppler information, thus simplifying the network structure. Recent studies have investigated Two-Stream Fusion Networks as potential solutions for challenges in this field. Tu et al. [29] created the “Joint-bone Fusion Graph Convolutional Network”, a novel model that leverages both skeletal and joint data to improve semi-supervised skeleton action recognition. C. Dai et al. [30] highlighted the importance of temporal dynamics in human action recognition through a two-stream attention-based LSTM network. The inclusion of attention mechanisms in the two-stream architecture demonstrated the network’s ability to enhance recognition accuracy. However, RNNs are commonly employed in previous studies, which fail to fully harness the parallel computing capabilities of GPUs to improve computational efficiency.

In this paper, we deploy a 77 GHz FMCW multiple-input multiple-output (MIMO) radar to capture radar echoes and propose a Deformable Dual-flow (DDF-CT) network based on CNN and temporal convolutional networks (TCN) for gesture recognition. The DDF-CT network provides more information for comprehensive feature extraction in gesture recognition by integrating time-frequency images and spectrogram sequences. We collect 1800 samples from six volunteers, comprising six types of gestures in a laboratory environment with random interferences, for model training, parameter tuning, and testing. Additionally, we amass 300 samples of the same six gestures in a different meeting room as test sets to demonstrate the adaptability of our method across different scenarios. The raw radar signals are converted to RTM and range-angle map sequences (RAMS) to reduce the impact of environmental changes. Robust features are extracted through 2D deformable convolutions, an inter-frame attention mechanism is incorporated into the TCN to learn the correlation among different time points within the gesture map. Unlike RNNs, the TCN performs parallel computing to process the sequence data, thus significantly improving the computational efficiency and achieving more stable gradient propagation. The in-depth analysis of the data and model structure shows that our method has outperformed other existing gesture recognition methods, achieving an accuracy level of 98.61% in the original environment and an accuracy level of 97.22% in a new environment.

## 2. Signal Processing

### 2.1. Principle of the FMCW Radar

The FMCW radar measures the frequency and phase differences between the transmitted and received signals, thereby accurately identifying the target object’s range, Doppler shift, and angle.

A transmitted signal of the FMCW radar can be expressed as

(1) $$S_{T}\left(t\right)=\cos\left(2\pi \left(f_{0}t+\frac{B}{2T}t^{2}\right)\right)$$

where $f_{0}t$ is the initial frequency, *B* is the bandwidth, and *T* is the scan time.

The transmitted signal reflects off the target and returns to the radar receiver after a time delay. The received signal and time delay can be defined as

(2) $$S_{R}\left(t\right)=\cos\left(2\pi \left(f_{0}\left(t-\tau \right)+\frac{B}{2T}{\left(t-\tau \right)}^{2}\right)\right)$$

(3) τ=2(R−νt)/c

where *R* is the range between the target and the radar, *v* is the relative velocity of the target, and *c* is the speed of light.

The transmitted and received signals are mixed to obtain an intermediate frequency (IF) signal. The mixed signal contains high-frequency components, which are removed using a low-pass filter, and the remaining signal is the IF signal. The IF signal and the beat frequency can be represented as

(4) $$S\left(t\right)=\cos(2\pi \left(\frac{2BR}{Tc}-\frac{2\nu }{c}f_{0}\right)t+f_{0}\frac{2R}{c})$$

(5) $$f_{b}=\frac{2BR}{Tc}-\frac{2\nu }{c}f_{0}$$

The IF signal at the $n^{th}$ sampling point of the $l^{th}$ chirp received by the $k^{th}$ antenna in a multi-receiver antenna setup can be expressed as

(6) $$S\left(n,l,k\right)=\cos(2\pi \left(\frac{2B(R+\nu Tl)}{Tc}-\frac{2v}{c}f_{0}\right)\frac{T}{N}n+f_{0}\frac{2(R+\nu Tl)}{c}+f_{0}\frac{kd\sin(\theta )}{c})$$

where *N* is the number of samples in one chirp, and *d* is the distance between adjacent receiver antennas.

According to Equation (6), the raw signal is a data cube consisting of ADC samples, chirps, and receiver antennas. Hence, the range, velocity, and angle of the gesture can be obtained by performing a 3D-FFT operation on the raw signal.

(1) 1D-FFT

According to Equation (6), the range *R* can be calculated as follows:

(7) $$R=\left(f_{b}+\frac{2\nu }{c}f_{0}\right)\frac{Tc}{2B}$$

On this basis, a 1D-FFT operation can be performed along the axis of ADC samples to estimate the beat frequency and compute the target range.

(2) 2D-FFT

A frame consisting of *L* chirps is established to determine the velocity of a target object. From the phase difference (Δφ) caused by the Doppler effect between two adjacent chirps, the velocity of the target object is derived as follows:

(8) $$\nu =\frac{\lambda \Delta \varphi }{4\pi T}$$

By performing an FFT (2D-FFT) operation along the chirp axis, the phase shift can be determined, and the velocity of the target object can be calculated.

(3) 3D-FFT

The angle of arrival (AoA) can be calculated from the phase change between adjacent receiver antennas. If there is a phase difference (*w*) between two adjacent receiver antennas, the AoA can be calculated as follows:

(9) $$w=\frac{2\pi d\sin(\theta )}{\lambda }$$

(10) $$\theta =\arcsin\left(\frac{w\lambda }{2\pi d}\right)$$

By performing an FFT (3D-FFT) operation along the axis of the receiver antenna, the phase difference *w* can be obtained, and the AoA can be calculated.

### 2.2. Acquisition of Datasets

**Figure 1:**
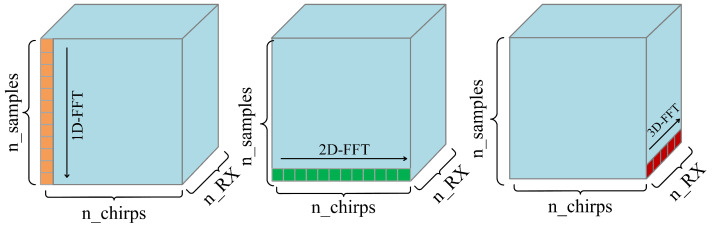
Processing of radar data cubes by FFT.

As shown in Figure 1, each frame of raw radar data forms a cube comprising ADC samples, chirps, and receiver antennas. To estimate the target distance, FFT operations are performed on all samples (marked in yellow) of each chirp. To estimate the target velocity, 2D-FFT operations are performed all chirps (marked in green) of each antenna. For angle estimation, 3D-FFT operations are performed on all receiver antennas (marked in red).

However, after relevant information is extracted through FFT, practical applications require further processing to remove clutter. For RTMs, the static noise is suppressed by calculating the difference between chirps and applying a window function. In order to obtain RAMS with less noise, a noise reduction algorithm was developed.

Since the Doppler frequency can distinguish between moving targets and static clutter, the Doppler frequency below the velocity threshold was set to zero to remove static clutter. To eliminate multipath reflections, we first summed values along the range dimension, calculating the total power for each angle bin. Then, based on a threshold determined by real-world conditions, bins with powers below the preset threshold were filtered out. In addition, the frame is recorded when the maximum value of RD is greater than the Doppler power threshold φ. To ensure reliable detection, data storage commenced once 15 consecutive frames satisfied the aforementioned conditions. The specific steps and parameters of this noise reduction approach are elaborated in Algorithm 1.
**Algorithm 1** Noise Reduction**Input:** Total number of frames: numFrame, FFT size: *L*, Doppler bin threshold: τ, scale factor of the angle bin power threshold: alpha, Doppler power threshold: φ, Range Doppler Matrix: **RD**, Range Angle Matrix: **RA****Output:** Range Angle Map Sequence: RAMS1:**for** i=1; i<numFrame; i++ **do**2:    Set $\mathrm{RD}(:,\frac{L}{2}-\tau :\frac{L}{2}+\tau )$ to 0;3:    Get the Doppler power of each angle bin **AP** by $AP=sum\left(\begin{array}{c} RA,1 \end{array}\right)$;4:    Get the Doppler power threshold *T* by $T=alpha\times \max\left(AP\right)$;5:    Initialize **RAMS** as null matrix and j=1;6:    **while** max(max(RD))>φ **do**7:        $\mathrm{RA}\left(:,\;\mathrm{AP}<\;T2\right)=\;0$;8:        RAMS(j,:,:)=RA;9:        j=j+1;10:        **if** j≥15 **then**11:           Save RAMS;12:        do end13:    while end14:for end

**Figure 2:**
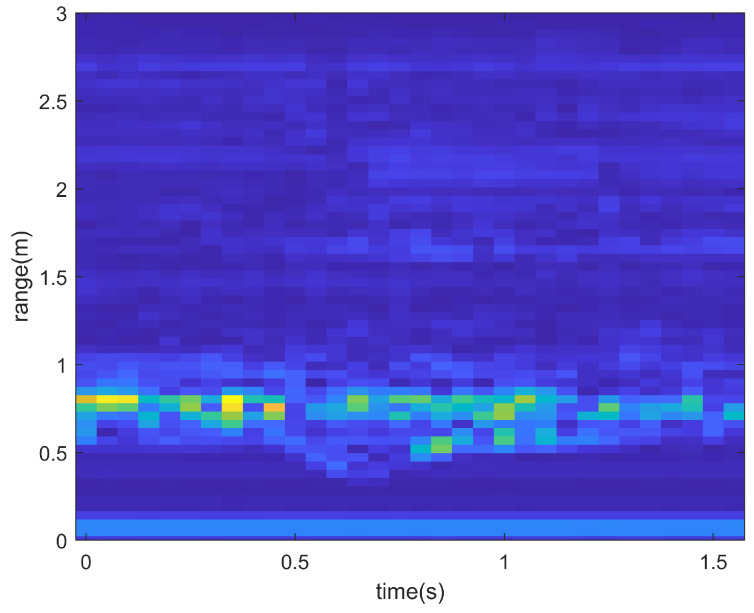
RTM for left swipe before noise reduction. In the RTM, pixel color, x-axis, and y-axis correspond to Doppler power, range, and time, respectively.

**Figure 3:**
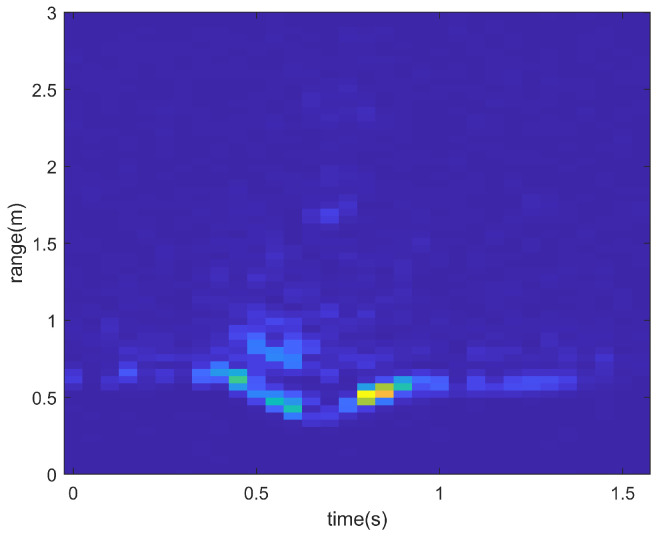
RTM for left swipe after noise reduction. In the RTM, pixel color, x-axis, and y-axis correspond to Doppler power, range, and time, respectively.

**Figure 4:**
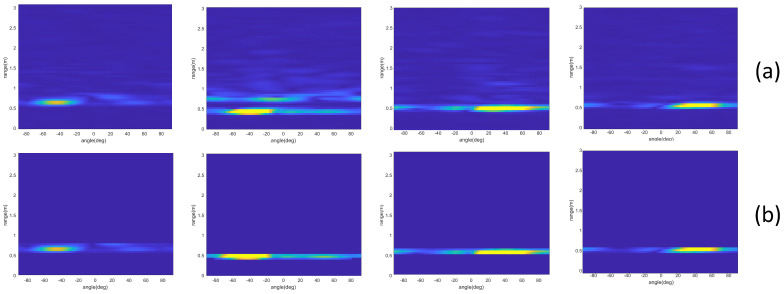
Comparison of RAMS before and after noise reduction. (**a**) RAMS for left swipe before noise reduction. (**b**) RAMS for left swipe after noise reduction. The rows represent the time series of four frames. In the RAMS, pixel color, x-axis, and y-axis correspond to Doppler power, range, and AoA, respectively.

Figure 2 and Figure 3 show the RTM when the user performs a left-swipe gesture. When the user performs a left-swipe gesture, the curve in the figure moves closer to the x-axis and then moves further away, which indicates a change in the distance of the hand. By comparing and analyzing Figure 2 and Figure 3, it can be seen that the static noise is significantly suppressed after differential computation. Figure 4 shows the RAMS for the same left-swipe gesture. During the left-swipe action by the user, the brightest section of the image shifts from left to right, indicating variations in AoA. The proximity to the x-axis first decreases and then increases, indicating variations in range. Comparing Figure 4a,b, it is evident that the features in the RAMS become more prominent and recognizable after the static clutter and noise are removed.

## 3. Proposed Network

**Figure 5:**
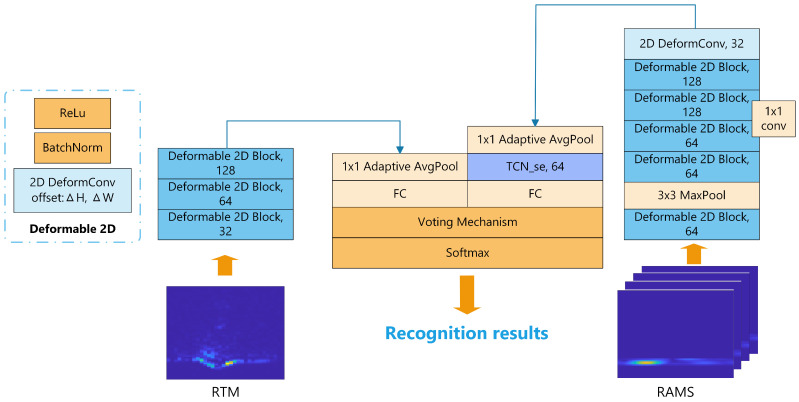
Structure of the DDF-CT network.

In computer vision, CNNs [31,32] have recently demonstrated a remarkable capability to derive spatial features from images autonomously. Meanwhile, TCNs [33] have emerged as a compelling alternative to LSTM networks for modeling time series data. With their inherent parallelism, TCNs ensure rapid processing of sequence data and exhibit enhanced stability during training. Moreover, the memory-efficient architecture of TCNs eliminates the need for storing hidden states for each time step like LSTM networks, and facilitates seamless end-to-end training. For these reasons, TCNs perform more consistently across various datasets and can alleviate the intricacies commonly encountered during the training phase of recurrent networks such as LSTM networks. Therefore, we design a novel network based on CNN-TCN dual-stream fusion, which consists of deformable convolution and inter-frame attention mechanisms. The overall architecture of the proposed DDF-CT network is shown in Figure 5.

**Figure 6:**
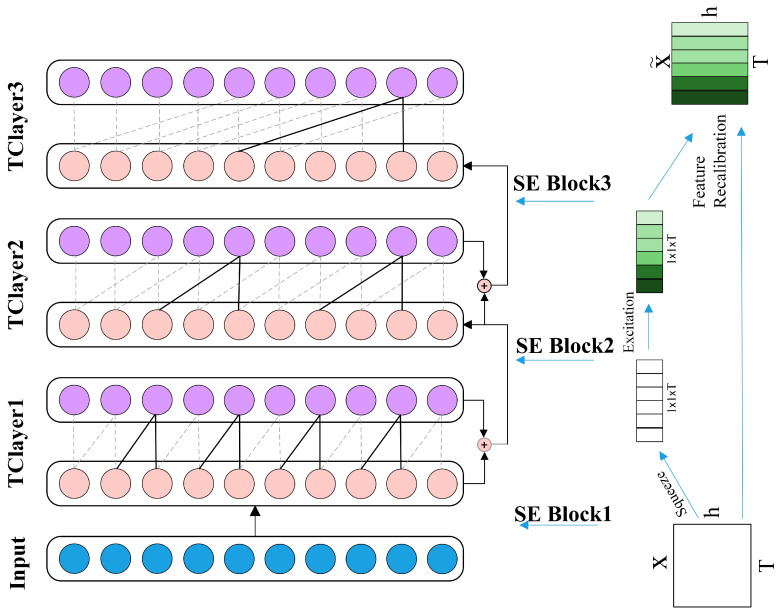
Structure of the TCN_se.

(1) Dual-stream network: The TFM and SMS are combined to overcome the limitations of singular representations of the data. Single-flow networks are not suitable for learning the joint data of maps and sequences. As a solution, the designed DDF-CT network is introduced to process the aggregated data simultaneously and thereby enable comprehensive feature extraction for hand gesture recognition (HGR). The network extracts spatial features from each RAM through a CNN and then uses a TCN to learn the temporal correlation within the entire sequence. In the meantime, employing a deformable convolution-based CNN, features are extracted from RTM. Finally, a voting mechanism is used to merge the outputs of dual streams to obtain a final classification decision.

(2) Deformable convolution: Variations in feature maps arise due to differences among users and environmental conditions. To address this, we adopted the deformable convolution module, inspired by its mechanism of adaptive deformation control over irregular offsets [34]. This choice replaces the original input convolutional layer, enabling the network to automatically adapt to changes in the shape and scale of feature maps, thereby enhancing the robustness of HGR. Within this framework, input features are processed by a specialized convolutional layer that not only extracts feature maps, but also predicts offsets for each spatial location. These offsets deviate from the kernel’s regular grid, facilitating adaptive kernel reshaping. Once generated, these offsets are integrated into the convolutional kernel, inducing its deformation. This adaptability allows the kernel to discern irregular patterns in the input, potentially missed by standard kernels. Importantly, these offsets are learnable, being refined during training for optimal data alignment. By leveraging these learned offsets, the network can simulate diverse geometric transformations, which is essential in gesture recognition to consider individual and situational differences.

(3) Inter-frame attention mechanism: Inspired by Squeeze-and-Excitation Networks [35] (SEnets), we use 32 frames of RAMS data as the inputs of 32 channels. Figure 6 shows the structure of the TCN_se. In the SEnet, the spatial information is first compressed through adaptive average pooling to obtain the global features of each channel. Then, the inter-channel correlations are extracted by a fully connected layer, and a sigmoid function assigns a corresponding weight to each channel. These weights are then multiplied by the original features to obtain weighted features that highlight the most critical channel information. After such processing, it is possible to detect the correlations among the 32 frames of data more accurately and efficiently extract relevant information from the key frames, which can significantly improve the accuracy of gesture recognition.

## 4. Experiment and Analysis

### 4.1. Experimental Platform

The experimental platform consists of the AWR1642 mm-wave radar sensor from Texas Instruments (TI) and the DCA1000 EVM data acquisition card. The parameters of the radar system are listed in Table 1. The frame rate, range resolution, and velocity resolution of the radar are 20 fps, 0.047 m, and 0.039 m/s, respectively. By using the MIMO radar technique, we have obtained an angular resolution of about 15. All experiments were conducted at a workstation with NVIDIA GTX3090 GPU and 3.3 GHz Intel i9-10940X CPU.

### 4.2. Dataset

**Figure 7:**
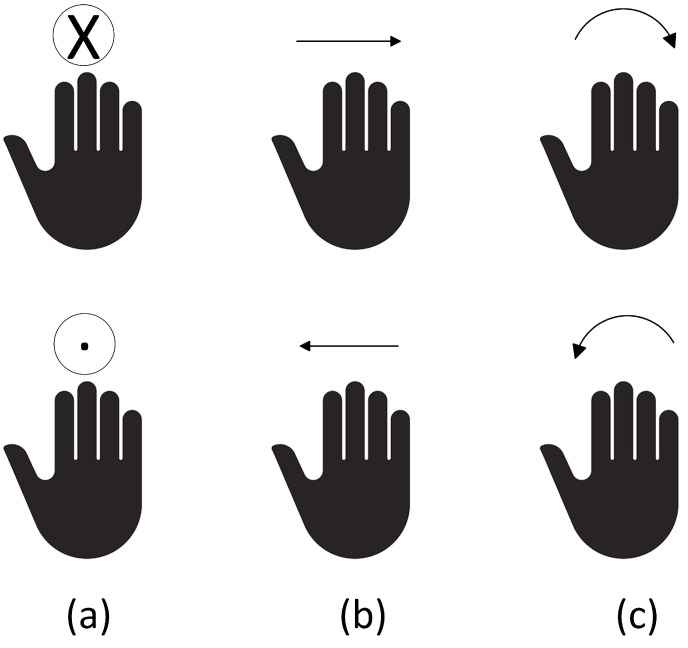
Dynamic hand gestures. (**a**) PH and PL. (**b**) RS and LS. (**c**) CT and AT.

To evaluate the effectiveness of our HGR system, we collected 1800 samples representing six gestures in a laboratory setting with random interferences. It can be seen from Figure 7 that these gestures are pulling (PL), pushing (PH), left swipe (LS), right swipe (RS), clockwise turning (CT), and anticlockwise turning (AT), all of which are easy to remember and perform. We asked six participants to perform each gesture 50 times according to their habits while ensuring that the distance between the radar and each participant ranges from 0.1 m to 1.5 m. In addition, we collected 50 samples for each gesture from five participants in a conference room and used these samples to test the adaptability of the system in new environments. Both environments (the laboratory and the conference room) were set up in their usual arrangements without any specific modifications for the experiment. During the data collection process, apart from the individual performing the gestures, other personnel might randomly appear on the scene. This simulates real-world scenarios, ensuring the universality and robustness of our model. To minimize background noise and reduce the computational load of the neural network, we retained only the first 32 range bins, namely, the range bins within 1.5 m. The angle-FFT was set to 32. Therefore, the sizes of both RAMs and RTMs are 32 × 32. Then, we cropped or added all the gestures movements to 32 frames. The final size of the RAMS is 32 × 32 × 32.

In this work, we divided our dataset into training, validation, and test subsets, accounting for 70%, 10%, and 20% of the data, respectively. The Adam optimization algorithm was used to train the proposed DDF-CT network at an initial learning rate of 0.01. The loss was computed using a cross-entropy function. To improve training dynamics, a learning rate scheduling strategy was implemented. The scheduling strategy is as follows: if the validation loss does not improve over five consecutive epochs, the learning rate is reduced to 10% of its current value.

### 4.3. Ablation Studies

To evaluate the performance of each block in the proposed network, the RAMS and RT dataset were selected, respectively, and adopted to verify the role of deformable convolution and inter-frame attention mechanisms. Therefore, the model mentioned below contains only the SMS flow component.

**Figure 8:**
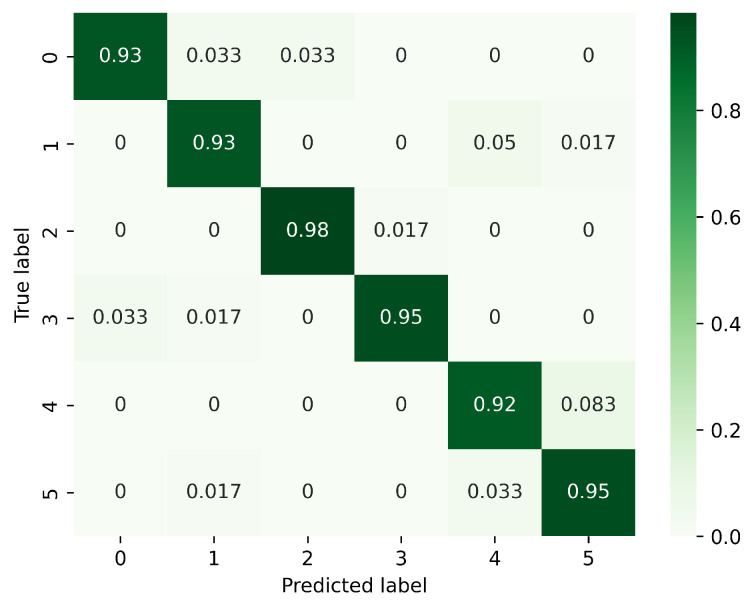
Confusion matrix for the model without DeformConv and SEnet. (0: PH, 1: PL, 2: LS, 3: RS, 4: CT, 5: AT).

**Figure 9:**
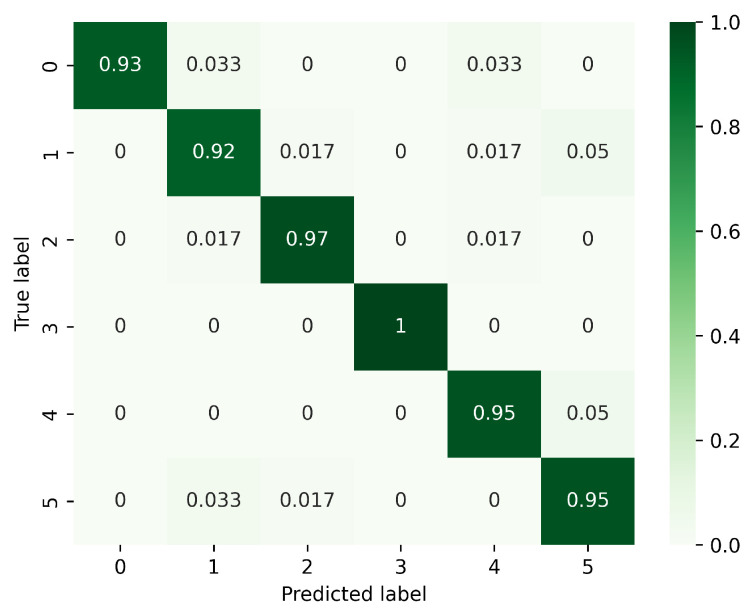
Confusion matrix for the model with DeformConv. (0: PH, 1: PL, 2: LS, 3: RS, 4: CT, 5: AT).

**Figure 10:**
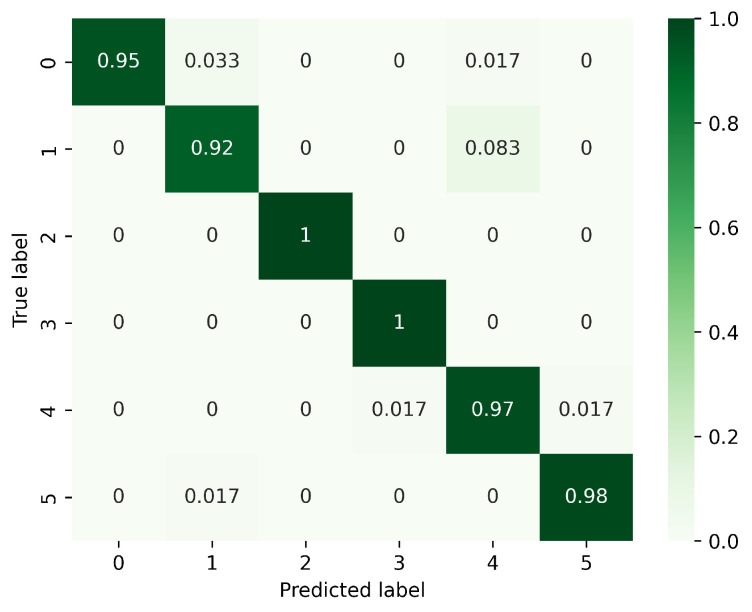
Confusion matrix for the model with DeformConv and SEnet. (0: PH, 1: PL, 2: LS, 3: RS, 4: CT, 5: AT).

Figure 8, Figure 9 and Figure 10 present the confusion matrices corresponding to different models, focusing on the analysis of gesture recognition accuracy with the integration of various modules. In these figures, 0 corresponds to PH, 1 to PL, 2 to LS, 3 to RS, 4 to CT, and 5 to AT. Figure 8 displays the confusion matrix for the model without DeformConv and SEnet. In this model, the accuracy rate for each gesture reaches over 90%. Figure 9 illustrates the confusion matrix for the model incorporating DeformConv. Notably, upon integrating deformable convolutions, the accuracy of RS has been improved remarkably. Figure 10 portrays the confusion matrix for the model with both DeformConv and SEnet. Not only does it sustain the enhancement in RS gesture accuracy, but the subsequent incorporation of inter-frame attention mechanisms also further improves the gesture recognition performance for RS, LS, CT, and AT.

Table 2 shows a 0.83% increase in average accuracy after the incorporation of deformable convolutional blocks and a 2.5% increase in average accuracy after the incorporation of an additional inter-frame attention mechanism. In the original CNN-TCN architecture, critical and non-critical feature regions are equally involved in gesture classification, which prevents the accuracy of gesture recognition from being further improved. To address this issue, deformable convolution and inter-frame attention mechanisms have been introduced. Deformable convolution enables the network to handle spatial variations, which improves the network’s ability to recognize irregular gesture patterns. Meanwhile, SEnet makes the network more inclined to emphasize the crucial features and reduces the interference of secondary information. The combination of these two strategies enables the network to perform gesture recognition tasks more accurately, resulting in an accuracy gain of 2.5%.

Since the TFM stream does not incorporate attention mechanisms, we only compared the basic convolutional network with the improved deformable convolutions. Table 3 shows a 2.06% increase in average accuracy after the incorporation of deformable convolutional blocks.

### 4.4. Comparison of Different Methods

In order to validate the effectiveness of the proposed method, four models, namely, 3D-CNN, CNN-GRU, CNN-LSTM, and CNN-BiGRU, were selected and compared with the SMS stream of the DDF-CT network (CNN-TCN with DeformConv and SEnet).

**Figure 11:**
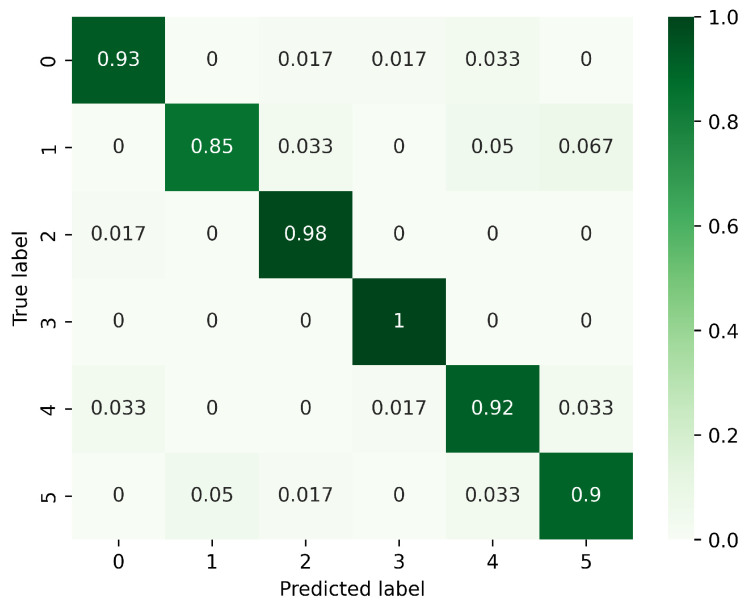
Confusion matrix for CNN-LSTM. (0: PH, 1: PL, 2: LS, 3: RS, 4: CT, 5: AT).

**Figure 12:**
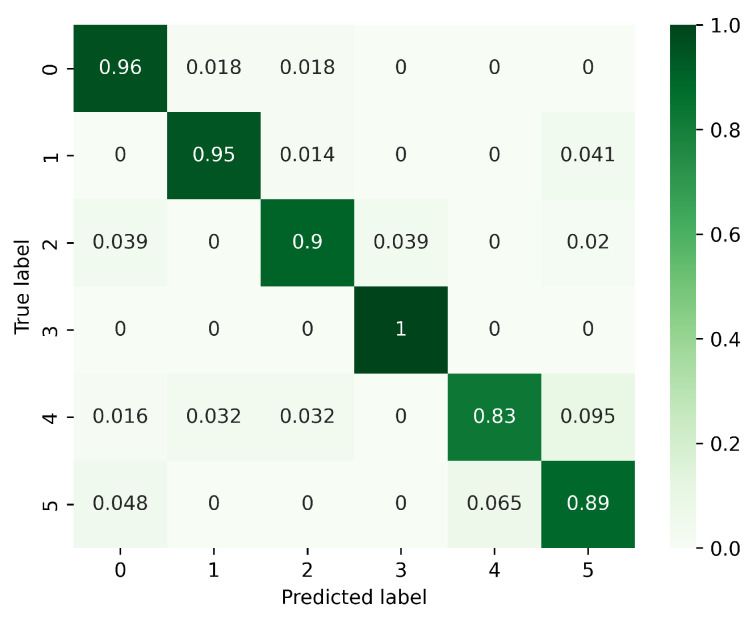
Confusion matrix for CNN-BiGRU. (0: PH, 1: PL, 2: LS, 3: RS, 4: CT, 5: AT).

Table 4 demonstrates that the DDF-CT network has a significant advantage over other networks in terms of accuracy, due to its ability to recognize and process key features more efficiently. From Figure 11 and Figure 12, it can be discerned that the accuracy rate for the PL and AT action using the CNN-LSTM method is notably low. Similarly, the CNN-BiGRU method exhibits a significant decrease in accuracy for recognizing CT, LS, and AT actions.

### 4.5. Comparison of Different Inputs

**Figure 13:**
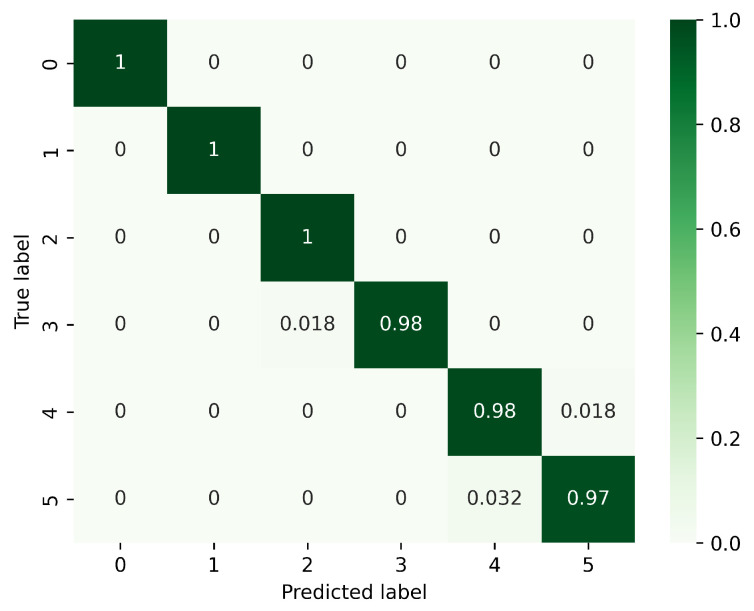
Confusion matrix for the DDF-CT network (0: PH, 1: PL, 2: LS, 3: RS, 4: CT, 5: AT).

To assess the contributions of different datasets to gesture classification, the RTM, RAMS, and combined datasets were input into the TFM stream of the DDF-CT network, the SMS stream of the DDF-CT network, and the entire DDF-CT, respectively. The outcomes of these experiments are presented in Table 5. The experimental results show that the accuracy of the dual-stream fusion model is 98.61%, which is higher than that of the single-stream network. Dual-stream data combine spatial richness with temporal ordering, which can provide a comprehensive view of the gesture to be recognized, thus improving the accuracy of gesture recognition. In addition, the performance of RTMs lacks the angular insights provided by RAMS. Figure 13 shows the confusion matrix for the DDF-CT network. From Figure 13, it can be seen that the DDF-CT network can recognize all gestures with high accuracy.

### 4.6. Testing in a New Environment

To validate the adaptability of our model in unfamiliar settings, 300 datasets containing 50 samples for each gesture were gathered in a new conference room. The findings are presented in Table 6. The DDF-CT network proposed herein exhibits robust adaptability and outperforms other models in novel environments.

## 5. Conclusions

In this paper, a new DDF-CT network is proposed to improve the robustness of HGR, which performs well in new environments. First, the radar echoes are preprocessed and denoised to construct the RTM and RAMS datasets. By establishing a dual-stream fusion model, these two types of gesture data and deformable convolution and inter-frame attention mechanisms are further utilized to improve the accuracy and stability of the algorithm. The experiment results show that the proposed method has achieved an accuracy level of 98.61% in the original environment and 97.22% in the new environment, both of which are significantly higher than those of other methods.

## Figures and Tables

**Table 1:** Parameters of the radar system.

Parameter	Value
Number of transmitter antennas	2
Number of receiver antennas	4
Frame periodicity	50 ms
Total bandwidth	3999.48 MHz
Number of sample points	128
number of chirps in one frame	128

**Table 2:** Gesture recognition results of methods with different structures added.

Model	Dataset	Accuracy (%)
CNN-TCN	RAMS	94.44
CNN-TCN with DeformConv	RAMS	95.27
CNN-TCN with DeformConv and SEnet	RAMS	96.94

**Table 3:** Gesture recognition results of methods with different networks.

Model	Dataset	Accuracy (%)
CNN	RT	91.77
CNN with deformConv	RT	93.83

**Table 4:** Gesture recognition results for different models.

Model	Accuracy (%)
3D-CNN	84.16
CNN-GRU	90.55
CNN-LSTM	93.05
CNN-BiGRU	91.94
Ours	98.61

**Table 5:** Gesture recognition results for different datasets.

Model	Dataset	Accuracy (%)
TFM stream of the DDF-CT network	RT	93.83
SMS stream of the DDF-CT network	RAMS	96.94
Entire DDF-CT network	RAMS + RT	98.61

**Table 6:** Accuracy of new environment test.

Model	Accuracy (%)
3D-CNN	79.00
CNN-GRU	84.33
CNN-LSTM	86.66
CNN-BiGRU	82.66
DDF-CT network	97.22

## Data Availability

The data and the code of this study are available from the first author upon request.
